# Protocol for a randomised controlled trial of cognitive bias modification training during inpatient withdrawal from alcohol use disorder

**DOI:** 10.1186/s13063-018-2999-3

**Published:** 2018-11-01

**Authors:** Victoria Manning, Joshua B. B. Garfield, Samuel C. Campbell, John Reynolds, Petra K. Staiger, Jarrad A. G. Lum, Kate Hall, Reinout W. Wiers, Dan I. Lubman, Antonio Verdejo-Garcia

**Affiliations:** 1 Turning Point, Eastern Health, 110 Church Street, Richmond, VIC 3121 Australia; 2 Eastern Health Clinical School, Monash University, Melbourne, VIC Australia; 3 Alfred Health and Faculty of Medicine, Nursing and Health Sciences, Monash University, 553 St Kilda Road, Melbourne, VIC 3004 Australia; 4 School of Psychology, Deakin University, Locked bag, Geelong, VIC 2200 Australia; 5 Addiction Development and Psychopathology (ADAPT)-lab, Department of Psychology, University of Amsterdam, PB 15916, 1001 NK Amsterdam, Netherlands; 6 School of Psychological Sciences and Monash Institute of Cognitive and Clinical Neurosciences (MICCN), Monash University, 18 Innovation Walk, Clayton Campus, Wellington Road, Clayton, VIC 3800 Australia

**Keywords:** Alcohol use disorder, Cognitive bias, Approach bias, Alcohol withdrawal treatment, Relapse, Abstinence, Cognitive training

## Abstract

**Background:**

People with alcohol use disorders often exhibit an “alcohol approach bias”, the automatically triggered action tendency to approach alcohol. Approach bias is likely to persist following withdrawal from alcohol, and contribute to the high rate of relapse following withdrawal treatment. Cognitive bias modification (CBM) training has been shown to attenuate approach biases and lead to reduced relapse rates. However, no large multisite trial of CBM specifically within a residential withdrawal treatment setting has previously been conducted. This study aims to test whether CBM delivered during residential withdrawal treatment leads to reduced relapse rates and reduced use of acute health services following discharge, and to test possible moderators of CBM’s effect on alcohol use.

**Methods:**

Three hundred alcohol-dependent inpatients are being recruited from three withdrawal treatment units in the Melbourne metropolitan area. Participants complete baseline measures of alcohol approach bias and cue-evoked desire for alcohol, followed by four daily sessions of computerised CBM training (or sham training if randomised to the control group). Approach bias and cue-evoked desire are re-assessed following the fourth training session. Follow-up assessments administered 2 weeks and 3, 6, and 12 months following discharge from the withdrawal treatment unit compare abstinence rates and acute and emergency healthcare service use between conditions. Pre-admission and follow-up substance use is derived from the timeline follow-back method, and approach bias towards alcohol with a computerised Approach Avoidance Task.

**Discussion:**

This study is the first multisite randomised controlled trial of cognitive bias modification delivered during acute alcohol withdrawal treatment. Withdrawal is theoretically an ideal period to deliver neurocognitive interventions due to heightened neuroplasticity and cognitive recovery. If effective, the low cost and easy implementation of CBM training means it could be widely used as a standard part of alcohol withdrawal treatment to improve treatment outcomes. Moderation analyses may help better determine whether certain subgroups of patients are most likely to benefit from it and therefore should be prioritised for CBM during alcohol withdrawal treatment.

**Trial Registration:**

Version 4 of the protocol (dated 1 August 2017) is registered with the Australian New Zealand Clinical Trials Registry, ACTRN12617001241325. Registered on 25 August 2017 (retrospectively registered).

## Background

Alcohol is one of the world’s most harmful drugs [1], responsible for 3.6% of deaths globally and 4.5% of the global burden of disease [2]. Alcohol consumption is a causal contributor to eight different cancers, numerous cardiovascular disorders (including hypertension, haemorrhagic stroke, and atrial fibrillation), pancreatitis, several liver diseases (notably cirrhosis and alcoholic hepatitis) and diabetes [3]. Excessive use can cause severe structural and functional neural abnormalities and result in significant cognitive dysfunction [4–6].

In Australia, alcohol is the most widely used drug, aside from caffeine. Despite recent evidence that people are drinking alcohol less frequently and more are choosing to abstain in recent years, many Australians continue to drink heavily and experience alcohol-related harm [7, 8]. Statistics indicate that more than one fifth of Australians meet lifetime *Diagnostic and Statistical Manual of Mental Disorders, Fourth Edition* (DSM-IV) criteria for an alcohol use disorder (AUD) [9]. Standard treatment for severe alcohol dependence in Australia often involves costly, intensive inpatient withdrawal treatment to manage acute withdrawal symptoms, followed by outpatient counselling and/or alcohol pharmacotherapy. Approximately 90% of patients relapse after withdrawal treatment, many within days of discharge, preventing successful engagement with post-withdrawal treatment and necessitating further episodes of inpatient withdrawal treatment [10]. Repeated readmission to withdrawal treatment, combined with this patient group's high rate of acute health service use, puts a high burden on the healthcare system. Novel interventions that reduce the proportion of patients who relapse have the potential to deliver cost savings to health and social welfare systems, and benefit alcohol-dependent individuals’ health and wellbeing, as well as their families.

Contemporary models of addiction frame AUD as (at least partially) the result of faulty information processing systems [6, 11–13]. With chronic use, neuroadaptations in the striatum and limbic system (i.e. the “reward pathways”) drive automatic, impulsive, reward-seeking behaviour and induce sensitisation to alcohol and alcohol-related stimuli. This results in cognitive biases, including ‘attentional bias’, which is the tendency for alcohol cues to disproportionately capture attention, and ‘approach bias’, which is the tendency for alcohol cues to induce automatic approach actions [11, 14]. As individuals become more dependent on alcohol, these cognitive biases increasingly direct thought and behaviour towards alcohol cues in the environment, and it is suggested that the frontal-striatal executive system becomes less capable of moderating or suppressing the actions driven by the overactive striatal-limbic system. Some research has found that these cognitive biases positively predict hazardous drinking [15, 16], although these findings have not always been consistently replicated [17]. Some studies suggest that this relationship between cognitive bias and problematic drinking is moderated by personality traits, particularly impulsivity, although there are conflicting findings regarding whether impulsivity is related to stronger or weaker relationships between cognitive bias and drinking [18–20].

Cognitive biases are typically measured using computerised tasks involving responses (e.g. using a keyboard, joystick, or mouse) to motivationally relevant and neutral images [16]. Differences in reaction times when responding to motivational stimuli, relative to neutral stimuli, indicate cognitive bias. Cognitive biases can be modified by adapted ‘training’ versions of these measurement tasks, known as Cognitive Bias Modification (CBM) training [21, 22]. Early CBM centred on anxious and phobic patients, re-training them to focus on positive or neutral cues, rather than cues associated with threat or distress, and these techniques have since been successfully adapted for treatment of AUD [23].

In the alcohol Approach Avoidance Task (AAT), respondents are presented with both alcohol-related and non-alcohol-related images and asked to respond with either an approach or avoidance behaviour to an arbitrary component of the presentation (such as the orientation or framing of the image), using a joystick or another similarly interactive medium [24]. For example, the approach behaviour typically involves respondents pulling the joystick towards themselves, which increases the size of the image, approximating the physical experience of approaching the stimuli. Likewise, the avoidance behaviour typically involves pushing the joystick away, which reduces the size of the image, giving the appearance that it is ‘receding’ into the ‘distance’. Training involves pushing alcohol-related images away the majority of the time (e.g. typically 90–100%), so that respondents repeatedly practice an avoidance action that counters their bias to automatically approach these stimuli, thereby theoretically weakening the approach bias [22].

The first CBM program that specifically targeted alcohol approach bias in a clinical population involved four consecutive daily sessions of alcohol AAT training to alcohol-dependent inpatients who had recently (at least 3 weeks prior) completed clinically supervised withdrawal from alcohol [25]. At pre-test, all participants demonstrated a strong approach bias towards alcohol. At post-test, participants in the experimental treatment arm demonstrated an avoidance bias towards alcohol, whereas controls (including two groups: one who were ‘trained’ with a control version of the task that involved avoiding only 50% of the alcohol-related images, and approaching the other 50%; another who did not do any training at all) maintained their initial approach bias. Participants who received CBM also reported lower rates of relapse (defined as 3 or more consecutive days of drinking; 46%) than controls (59%) at follow-up 1 year after treatment discharge. In two large subsequent studies, it was found that 6 or 12 sessions of CBM training delivered later in the post-withdrawal inpatient program was also associated with improved rates of abstinence, although the effect size was slightly reduced relative to the earlier study (8.5% less relapse) [26, 27]. Subsequent functional magnetic resonance imaging (fMRI) studies have shown reduced activation in both the medial prefrontal cortex and the amygdala in response to alcohol cue presentation after training, which correlated with measured changes in approach bias [28, 29]. This suggests that CBM reduces cue-evoked brain activity in regions involved in motivational salience, which may explain the increased rates of abstinence observed after training.

Since these initial reports of the efficacy of CBM, additional studies have been published, allowing for systematic reviews and meta-analyses on the effectiveness of CBM. The first of these [30] cast scepticism on its effectiveness. However, this review conflated studies of attentional bias modification, approach bias modification, and modification of other types of cognitive biases; included multiple addiction populations (i.e. alcohol and tobacco); and included studies of both clinical and non-clinical (non-dependent) participants. The validity of the review’s conclusion has recently been challenged on the basis that CBM has differential effects in relation to study type (laboratory experiments with student populations versus randomised controlled trials [RCTs] in clinical populations), mode of delivery (in person in controlled conditions versus via internet) and population (treatment-seekers versus those not seeking to change their behaviour) [31]. The authors also note that in clinical settings in RCTs with alcohol-dependent patients, approach bias has modest but significant effects as an adjunct approach when delivered alongside other psychosocial interventions such as cognitive behavioural therapy. A recent systematic review focused specifically on approach bias modification studies for alcohol use, tobacco use, or unhealthy eating which included behavioural outcome measures concluded that it confers positive effects, in terms of reduced consumption and increased rates of abstinence [32].

During alcohol withdrawal, the brain undergoes extensive structural and functional recovery and reorganisation. Significant increases in the grey matter of the insular and anterior cingulate cortices have been detected within the first 2 weeks of withdrawal [33]. These areas are involved with interoceptive sensitivity and cognitive control and are integral to the operations of the reward and executive systems [34]. These structural repairs are thought to underlie improvements in cognitive functioning during and after withdrawal [35, 36] and are indicative of a period of heightened neuroplasticity that, if harnessed through cognitive training, may facilitate the amelioration of maladaptive cognitive biases.

For these reasons, we previously conducted a pilot study of four sessions of CBM delivered during inpatient alcohol withdrawal treatment [37]. This study of 83 patients found a near-significant increase in rates of abstinence during the first 2 weeks following discharge from the withdrawal unit, relative to a sham-training condition, and this effect was significant when analysis was restricted to those participants who completed all four sessions of training. We focused on outcomes during the first 2 weeks following discharge because this is a crucial time for commencing engagement in ongoing post-withdrawal treatment, which often fails to occur due to rapid relapse. However, this study was not adequately powered, particularly for examining abstinence rates at later follow-ups, and the control condition did not involve exposure to alcohol images, which may have confounded findings (due to potential exposure effects).

Aside from this small pilot study, previous studies of CBM in alcohol-dependent samples have waited until several weeks after withdrawal to commence training. The efficacy of CBM during withdrawal treatment has therefore not yet been tested in any large, multisite trials. The present study is thus the first large study of CBM in a residential withdrawal treatment setting. This will also allow analyses exploring the moderating effects of clinical and personality traits, particularly approach bias, impulsivity, cravings, and severity of dependence, to help further elucidate the mechanisms governing CBM’s efficacy and which specific types of patients are likely to benefit most from it. Moreover, it overcomes an important limitation of the previous pilot study by ensuring that participants in the control condition have equal exposure to the same images as those used in the CBM condition.

## Methods

### Aims

The primary objective is to determine the efficacy of CBM training, compared to sham training, in a population undergoing inpatient withdrawal treatment from alcohol, in terms of their abstinence rates at 2 weeks post-discharge.

The secondary objectives are:

1. To determine the efficacy of CBM training compared with sham training, in a population undergoing inpatient withdrawal treatment for AUD, in terms of their abstinence rates at 3, 6, and 12 months post-discharge.
2. To determine whether the efficacy of CBM compared to sham training is moderated by the strength of approach bias at baseline.
3. To determine whether the efficacy of CBM is moderated by risk-taking impulsivity.
4. To determine whether participants who undergo CBM training will demonstrate a reduction in self-reported cue-evoked desire (wanting) for alcohol compared to those who undergo sham training.
5. To determine whether those who receive CBM training impose a lower burden on healthcare services in terms of reduced use of emergency healthcare services and readmission to alcohol and other drug (AOD) withdrawal treatment services in the year following discharge, relative to those who receive sham training. These data will be used to estimate the cost savings to the healthcare system that would be associated with introducing CBM as a routine component of inpatient withdrawal treatment.

### Trial design

This is a randomised, double-blind, controlled, parallel-group trial. The protocol has been formulated in accordance with Good Clinical Practice, SPIRIT, and CONSORT 2013 guidelines.

### Study setting

Recruitment and data collection is taking place at three AOD residential withdrawal treatment units in the Melbourne metropolitan area in Australia.

### Sample size

Our recent pilot study indicated a difference in abstinence rates of 22% (i.e. 69% vs 47%) between the CBM and sham training groups. Using a smaller conjectured difference of 20% (e.g. 65% vs 45%) a two-sample, binomial test (two-sided α = 0.05) that utilises a pooled estimate of the variance, has 90% power to detect the conjectured difference when a total of 256 participants is randomised (i.e. 128 in each treatment arm). With an allowance for up to 15% drop out in the 2 weeks after discharge, based on an observed retention rate of 86% at 2 weeks in the pilot study, the target sample size was set at 300 (i.e. 150 randomised to each treatment arm). Recruitment is expected to take 22 months, given the throughput in the withdrawal treatment units, and the total study duration is likely to be 36 months.

### Eligibility criteria

**Fig. 1:**
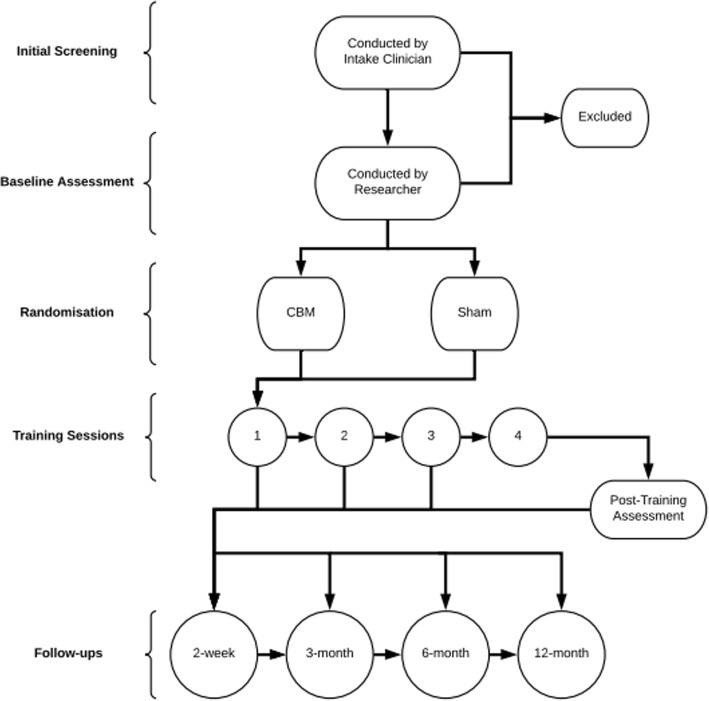
Participant flow through stages of the study

Participants must: be aged between 18 and 65; meet *Diagnostic and Statistical Manual of Mental Disorders, Fifth Edition* (DSM-5) criteria for moderate or severe AUD (i.e. at least four symptoms present in the past year); and have used alcohol at least weekly in the month prior to admission to inpatient withdrawal treatment. Patients are excluded from participation if they have a diagnosed history of neurological illness or injury, or any concussion resulting in a loss of consciousness longer than 30 min, or have any diagnosed intellectual disability. Patients assessed by withdrawal unit clinical staff to be unable to safely participate, or without capacity to provide informed consent, due to acute mental or physical impairment (e.g. uncontrolled physical or mental illness or withdrawal-related distress), are not approached for participation. Patients are ineligible to participate if they are currently participating in another clinical trial aiming to test and/or alter outcomes following discharge from inpatient withdrawal, or if they are not planning to stay in the inpatient withdrawal treatment unit long enough to complete four consecutive days of CBM training (See Fig. 1 for the CONSORT participant flowchart).

### Measures

#### Demographics

At baseline, a researcher administers a questionnaire assessing participants’ date of birth, identified gender, country of birth, Aboriginal or Torres Strait Islander status, education, relationship status, employment, housing, age of onset of alcohol use, age of onset of alcohol-related problems, number of prior withdrawal treatment episodes, presence of other drugs of concern, presence of substance use disorders among first-degree relatives, psychiatric diagnoses, and (to verify screening) presence of any brain injury or neurological disorders.

#### Alcohol use disorder severity

Alcohol use disorder symptoms are assessed at the baseline interview with the interviewer-administered alcohol use disorder module from the Structured Clinical Interview for DSM-5 Disorders – Research Version (SCID-5 RV), which also verifies eligibility [38]. Severity of physical dependence on alcohol is assessed with the self-administered Severity of Alcohol Dependence Questionnaire (SADQ) [39].

#### Recent alcohol and other substance use

The timeline follow-back (TLFB) interview method is used to quantify number of days of alcohol use and estimated standard drinks consumed [40]. At baseline, the TLFB covers the 30 days preceding admission to the inpatient withdrawal treatment unit. At the 2-week follow-up, the TLFB covers the time since discharge from the withdrawal treatment unit. At the 3-, 6-, and 12-month follow-ups, the TLFB covers the past 30 days. Researchers also use the TLFB to collect information on use of psychoactive medications, including pharmacotherapies for alcohol craving, tobacco and illicit drugs. Because the TLFBs at the 3-, 6-, and 12-month follow-ups do not cover the entire time since the previous follow-up, participants who have not yet lapsed (defined as any alcohol use) or relapsed (defined as 3 days in a row of consecutive alcohol use) at the previous follow-up are asked at the current follow-up whether there was any lapse or relapse since the previous follow-up and, if so, on what date it occurred.

#### Alcohol cravings

Alcohol cravings are assessed using the Alcohol Craving Questionnaire – Short Form – Revised (ACQ-SF-R) [41]. In addition, to monitor participant safety (related to the potential for exposure to alcohol-related imagery to trigger cravings), participants self-rate the intensity of their craving for alcohol immediately before and following each CBM training session, using a single-item visual analogue scale (anchored at the left and right ends with ‘not at all’ and ‘extreme’, respectively). In addition, participants view 20 computerised images (10 of alcoholic beverages and 10 of non-alcoholic beverages) and rate the magnitude of their ‘wanting’ of the pictured beverages by marking a point along an accompanying line with end caps either side indicating ‘I do not want this at all’ to ‘I really want this’. Scores range from 0 to 100, based on the position of the mark (i.e. a mark on the extreme left of the line results in a score of 0 being recorded, while a mark on the extreme right of the line results in a score of 100 being recorded). Within each of the two beverage categories (alcoholic; non-alcoholic), five of the 10 images are identical to images used in the training task (see below) and the other five are novel images not used in other study tasks. This is to allow assessment, following training, of generalisation of reduced cue-evoked wanting from images used in training to other alcohol-related images.

#### Service use

Use of AOD withdrawal treatment services, outpatient AOD counselling, AOD rehabilitation programs, general practitioner services, ambulance call-outs, emergency department visits, and hospital inpatient admissions are assessed with an interviewer-administered, modified version of the Lifetime Drug Use History Questionnaire (LDUH) [42]. At the baseline interview, these questions assess the year prior to the current inpatient withdrawal treatment admission. At each follow-up they assess the time since the previous follow-up (or since discharge in the case of the first follow-up since discharge, i.e. typically the 2-week follow-up), with data from the separate follow-ups then combined for each participant to form a composite assessment of service use over the whole 12-month follow-up period.

#### Approach bias

A modified version of the Alcohol-AAT is used to measure approach bias towards alcohol [24]. Participants are required to react to the format of pictures using a joystick (e.g. push landscape pictures, pull portrait pictures), irrespective of the content of the pictures. There are two categories of pictures (alcoholic beverages; non-alcoholic beverages, 10 unique pictures in each category). The images were selected to represent the beverage type and brands most commonly consumed by this population as documented in the recent pilot study [37]. Each image is repeated twice (for a total of 40 trials) and every picture type appears in landscape and in portrait format 50% of the time. For both categories of pictures (alcoholic; non-alcoholic), the median reaction time (RT) for pull responses is subtracted from the median RT for pull responses to calculate a measure of approach bias.

#### Risk-taking impulsivity

The Balloon Analogue Risk Task (BART) [43] is a validated behavioural measure of risk-taking impulsivity, in which participants are required to inflate a virtual balloon with ‘pumps’, each of which increases a small potential payout. However the balloon may randomly burst at any time, resulting in forfeiture of money earned for that trial. Fifteen trials are administered prior to commencing the first session of CBM. Mean-adjusted pumps (the average number of pumps per banked balloon) will be analysed as a measure of risk-taking impulsivity.

#### Participant assessments of training

**Fig. 2:**
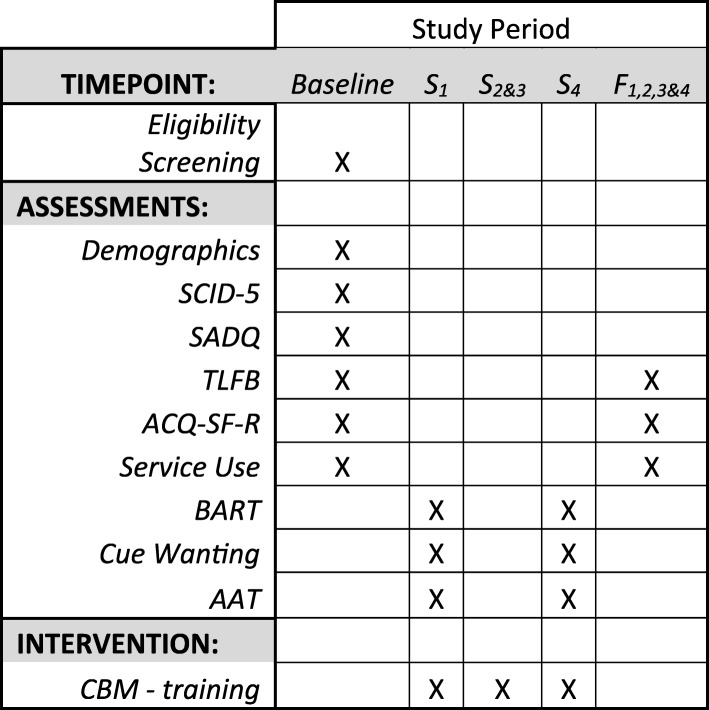
Schedule of measures and interventions. *AAT* approach avoidance task, *ACQ-SF-R* Alcohol Craving Questionnaire – short form – revised, *BART* Balloon Analogue Risk Task, *CBM* cognitive bias modification, *F* follow-up, *S* session, *SADQ* Severity of Alcohol Dependence Questionnaire, *SCID-5* Structured Clinical Interview for DSM-5 Disorders, *TLFB* timeline follow-back

After the final training session, participants are asked to rate the training program by reporting, using a five-point Likert-type scale (ranging from ‘strongly agree’ to ‘strongly disagree’), whether or not they believed that the training (i) improved their attention, (ii) reduced their craving for alcohol, and (iii) was interesting. See Fig. 2 for the schedule of measures and training sessions administered.

### Interventions

The CBM training is a modified training version of the assessment AAT. Participants are instructed to respond to digitally presented images with a push or pull motion, using a joystick, based on the orientation of the framing of the picture (pushing landscapes; pulling portraits). The training task has been programmed to scale up and down the images in response to pull and push movements, respectively, to simulate the picture expanding towards the participant when ‘pulled’ and receding into the distance when ‘pushed’. As a component of each training session, participants complete a brief practice round involving eight empty frames, to familiarise them with the task requirements. Following the eight practice trials, participants are exposed to presentations of 40 images of alcoholic and 40 images of non-alcoholic beverages, in a random order. Each alcoholic and non-alcoholic beverage is presented three times, for a total of 240 image presentations. Landscape-oriented frames contain alcoholic images in 95% of presentations, implicitly training participants to respond to alcohol with an avoidance movement. The remaining 5% of landscape-oriented presentations contain images of non-alcoholic beverages. Likewise, portrait-oriented images contain non-alcoholic images in 95% of presentations, and alcohol-related images the remaining 5% of the time. During each presentation, the image scales until the joystick reaches its maximal distance. If the response is correct, the next trial then commences after a 500 millisecond inter-stimulus interval (ISI). If the response is incorrect, a large red ‘X’ flashes on the screen and the trial is repeated until the correct response is performed.

The sham training is identical to the CBM training described above, except that each orientation (portrait or landscape) contains alcohol images 50% of the time and non-alcohol images the other 50% of the time. Moreover, instead of instructing participants to respond with approach or avoidance movements, participants are instructed to respond with lateral movements of the joystick, according to picture orientation (left for landscape; right for portrait). As in the experimental training, the image moves, in accordance to the joystick movement, to the left or right edge of the computer screen, at which point the next presentation begins after a 500 millisecond ISI (if the response was correct), or a red ‘X’ flashes and the presentation is repeated (if the response was incorrect). The pictures do not change size in this condition. The sham condition thereby controls for participants’ exposure to alcohol (and non-alcohol) images, and for the demand to attend to image orientation and manipulate the picture with a joystick based on orientation, without including the approach/avoidance component hypothesised to underlie the therapeutic effect of the AAT training.

### Allocation

Site-specific randomisation sequences were generated by a researcher not involved in recruitment or data collection using a random number generator prior to beginning recruitment. A site-stratified 1:1 treatment arm allocation ratio is used, and is based on permuted blocks of variable size. The allocation sequence for each site is programmed into the training task on that site’s task laptop. When opening the training task, the researcher administering the training is prompted to enter the participant’s number, which then automatically causes the program to select the CBM or sham training, based on the pre-programmed randomisation sequence, such that the participant is allocated into a treatment arm ‘automatically’ – with no input from a researcher and without the researcher being able to predict a participant’s allocation prior to randomisation.

### Procedure

Intake AOD clinicians at the participating withdrawal treatment units conduct preliminary screening of patients’ eligibility at admission, and briefly describe the study to patients who appear to meet the eligibility criteria. If patients express interest in participating, the clinician alerts the research team. A member of the research team approaches the patient no sooner than 2 days after their admission and provides them with a comprehensive verbal and written description of the study’s aims and procedures. The research team member obtains written consent if the patient is willing to participate. After the provision of informed consent, but prior to commencing CBM training, a researcher administers the baseline questionnaires to confirm eligibility and assess demographic and clinical characteristics (demographic questionnaire, SCID-5-RV, TLFB), facilitates participants’ self-administration of the ACQ-SF-R, and then proceeds to the computerised assessments (BART, picture wanting ratings, and approach bias measurement). To avoid participant fatigue, service use and SADQ questionnaires are occasionally delayed to the second or third day of training, but may also be administered on the first day if the participant prefers.

After they have completed the pre-training measures, participants are randomised into a treatment arm by the computer and begin their first training session. Training sessions continue on each of the next 3 days (i.e. 4 consecutive days of training in total). Following the final training session, participants repeat picture wanting ratings, the approach bias measurement task, ACQ-SF-R, and the three-item task rating. After their discharge from the withdrawal treatment unit, a researcher records the types, frequency, and dosages of any medications administered to participants during their admission, and makes copies of any relevant clinical notes taken at admission. Another researcher not involved with the participant’s training administration – and therefore blinded to their treatment allocation – contacts them at 2 weeks, 3, 6, and 12 months post-discharge to conduct telephone follow-up interviews consisting of the TLFB, service use questions, and ACQ-SF-R. Following intention-to-treat principles, participants who unexpectedly discharge from the inpatient withdrawal treatment unit prior to completing all four sessions of training are still contacted for follow-up, unless they withdraw from the research. Participants may withdraw at any time, and if they do, no further training or questionnaire measures are conducted. Participants are informed, prior to providing their consent to participate, that if they withdraw, their data collected prior to their withdrawal treatment will be retained for inclusion in analyses unless they explicitly rescind permission to retain these data.

### Outcome measures

The primary outcome of interest to this study is the difference between treatment arms in proportion of participants reporting abstinence from alcohol 2 weeks after discharge, measured with the TLFB. Secondary outcomes of interest are abstinence at the subsequent follow-ups (measured with the TLFB), the difference between treatment arms in healthcare costs incurred due to acute/emergency healthcare treatment and readmission to AOD withdrawal treatment during the follow-up period (service use measured with the LDUH, with costs of treatment episodes to be estimated with the assistance of a health economist), and differences in cue-evoked desire for images of alcohol after the final session of CBM training. Secondary analysis will also examine the degree to which baseline approach bias towards alcohol moderates the effectiveness of CBM training on the primary outcome. Additional exploratory analyses are planned to investigate (i) moderating effects of pre-admission history of withdrawal treatment, severity of alcohol dependence and cravings, and impulsivity on the primary and secondary outcomes; (ii) whether alcohol cravings (as measured by the ACQ-SF-R) are affected by CBM; and (iii) whether CBM affects time to relapse.

### Data management

Prior to analyses, data cleaning and verification will be conducted for all data entered manually (i.e. data collected using paper questionnaires). Range checks will be conducted on all fields to detect outliers and incorrect entries. Entries subsequently confirmed to be incorrect, after checking paper records, will be amended. Following this, a random sample of 10% of the cases from key fields will be re-entered. Where this results in discrepancies between the original data and the re-entered data, we will determine whether the original data was erroneous, and correct it if so. Using this method, any field that has a higher than 2% error rate will be completely re-entered.

### Statistical methods

The main analyses of the primary and key secondary outcome variables will follow the intention to treat (ITT) principle and will include all randomised patients regardless of completion of the training phase or loss to follow-up (the full analysis set). A supportive analysis of the primary outcome will be restricted to participants who completed all four training sessions and were subsequently assessed 2 weeks after discharge (the per protocol set).

### Primary outcome

In the ITT analysis of the primary outcome, the divisor for the proportion of abstinent participants in an arm will be the number randomized to that arm and individuals for whom alcohol use during the first 14 days post-discharge was not assessed, for any reason, will be deemed not to be abstinent. These proportions will be compared using a two-sample binomial test (two-sided α = 0.05) and a 95% confidence interval for the difference in the proportions will also be reported. The Cochran-Mantel-Haenszel test, stratified by site, will be conducted as a supportive analysis. In a further supportive analysis of the primary outcome, participants who complete fewer than four training sessions or who miss the 2-week assessment, will be excluded from the denominator (and the numerator) when the proportion of abstinent patients is calculated in each arm. This ‘per protocol’ analysis will use the same statistical methods as the ITT analysis.

### Key secondary outcome and moderation analysis

Assessments of abstinence in the 30 days prior to each of the 3-, 6- and 12-month follow-ups will also be analysed in the same way as the ITT analysis of the primary (2-week) endpoint – participants not assessed for any reason will be deemed to have relapsed. In a supplementary analysis of all available follow-up assessments (from 3 to 12 months), that assumes any missing follow-ups are missing completely at random, a logistic regression analysis, using the method of generalized estimating equations (GEE), will be used to compare the arms, and changes over time in the arms, adjusting, if need be, for sites. An additional supplementary, missing not at random (MNAR), analysis will use a Bayesian approach, and Markov chain Monte Carlo (MCMC) to jointly model abstinence and missingness. The model will include a random effect for each participant and minimally informative, normal prior distributions for parameters in the logistic models for abstinence and missingness. Parameter estimates and their associated 95% credible intervals, based on posterior distributions, will be reported. The MNAR approach will also be used to investigate the moderating effect of the baseline approach bias score. Additional exploratory analyses, also using the MNAR approach, will investigate adjusting the estimated difference between the arms for such covariates as age, gender, and SADQ score. Full details will be given in the statistical analysis plan (SAP) that will be documented prior to the analysis of the primary outcome.

### Other secondary outcomes

Other secondary outcomes that are binary will be analysed in the same way as the key secondary outcome measure, including supplementary analyses (as above). The moderating effect of baseline approach bias score on abstinence/relapse in the 2 weeks post discharge (i.e. the primary outcome) will be investigated by logistic regression models that include baseline scores as covariates in the model and tests of the significance of the two-way interaction of treatment arm with the covariate will be conducted. A similar approach will be used to test for a moderating effect of impulsivity, as measured by the BART.

Continuous-scale outcome measures, and ordinal scale outcomes that have five or more ordered categories, will be analysed using mixed models, and the restricted maximum likelihood (REML) method, with random effects for participants and assessments within participant, and fixed effects for treatment arm, time and baseline covariates. Diagnostic plots of residuals will be examined and, if required, analyses will be conducted using a variance-stabilising transformation such as the log transformation or the empirical logit transformation.

To determine the economic feasibility of CBM, in terms of savings to the treatment system (evidenced by fewer repeat inpatient withdrawal treatment episodes and episodes of acute health service use at the 12-month follow-up), we will compare net spending (cost of CBM intervention plus cost of further withdrawal/acute health service use for each participant) in the CBM group to net spending (cost of further withdrawal/acute health service use) in the control group. The statistical significance of the difference between the groups will be assessed with a *t* test and a variance-stabilising transformation, such as the logarithm, will likely be required.

For cue-evoked wanting, outcomes will be assessed with a mixed model (repeated measures) analysis assessing within-subjects variables of ‘time’ (pre-training/post-training), ‘picture-type’ (alcohol/non-alcohol), and ‘novelty’ (used in training/not used in training), with the between-subjects conditions of ‘group’ (CBM/Control). Particular attention will be paid to comparisons of time, picture-type, and group within each level of novelty. The number and proportion of participants who require cessation of one or more training sessions due to distress or fear of relapse, and who require permanent cessation of training for these reasons, will be reported as a measure of the safety of the CBM training. Statistical analyses will be conducted using the most appropriate procedures in GenStat, 19th Edition (VSN International, Hemel Hempstead, UK), SAS Version 9.4 (SAS Institute, Inc., Cary, NC, USA) and R (R Foundation for Statistical Computing, Vienna, Austria), or later versions of the software as they become available.

## Discussion

This study is the first multisite randomised controlled trial of cognitive bias modification as a stand-alone intervention delivered during residential alcohol withdrawal treatment. Alcohol withdrawal is a period of extensive neural and cognitive recovery [33, 35, 36]. This could therefore be an opportune context in which to retrain the automatic approach tendency towards alcohol that is characteristic of alcohol use disorder and, importantly, is believed to help drive the high rates of relapse in this population. This study also has the potential to inform the development of approach bias modification training programs for patients withdrawing from other substances, such as illicit drugs.

If found to be effective, CBM could serve as a minimally invasive, cost-effective, easily adopted adjunctive treatment that can help extend the period of abstinence after being discharged from inpatient withdrawal treatment. This could lead to considerable cost savings to the health system by reducing repeated readmissions to withdrawal treatment and by reducing emergency health service use due to alcohol-related injury and illness. Additionally, by examining potential moderating factors, the results would expand the extant literature on who benefits most from CBM and who is best targeted during withdrawal. The examination of changes in wanting (motivational salience of alcohol cues) has the potential to shed light on the underlying mechanisms of CBM.

There are a range of practical and operational issues associated with conducting this study, mainly arising from the characteristics of this population and of alcohol withdrawal treatment. Participants are recruited on the understanding that it will be possible to run the intervention according to the protocol (i.e. that there are at least 4 consecutive days of inpatient treatment on which to run CBM training sessions prior to the planned date of discharge). However, clients’ length of stay in a withdrawal treatment setting and day-to-day wellbeing can be subject to unexpected change for a variety of reasons, preventing completion of four sessions on 4 consecutive days. Where a participant is unwell or unavailable on a day on which a session was planned, we continue training on the following day if possible. In some cases, this means that non-completion of the four-session protocol prior to discharge is unavoidable, however adjustments we consider acceptable for the purpose of this study include administering two sessions (morning and afternoon) on one day to ‘catch up’ when a day has been skipped, or if the participant’s planned date of discharge is moved earlier such that four sessions could no longer be completed if only one session was done per day. In addition, participants are in alcohol withdrawal, are often medicated with sedating drugs, and the highly structured program of activities that withdrawal treatment facilities require patients to participate in limit time available for CBM training. Thus, excising some of the secondary measures due to participant fatigue, distress, or lack of time, is sometimes necessary to ensure completion of the core CBM training protocol and completion of the most necessary measures.

Instability in residential status, relationships and employment is common among this population, and this makes follow-up assessment particularly challenging. In our pilot study of CBM training [37], 86% of the sample completed the 2-week follow-up. Based on previous research [44], we anticipate retention of approximately 70% of participants at the 12-month follow-up. To support these targets, during the course of recruitment and consent, participants are asked to provide several means of contact, such as home and mobile phone numbers, email, postal addresses, and multiple secondary contacts, such as family members, friends, or significant others. Researchers conducting follow-ups are available to contact participants at any time of their choosing, and participants are reimbursed AUD 10 for completing each of the follow-ups. At each follow-up, participants are reminded of any upcoming follow-ups to minimise attrition.

Due to the nature of the training task (exposure to images of alcohol), it is expected that participation will trigger cravings or distress in some participants. To monitor this, and minimise adverse consequences for the participants, participants rate their craving for alcohol immediately prior to and following administration of each training session. If a participant reports strong cravings following a session, researchers offer information on a mindfulness-based technique developed to aid patients in recovery manage instances of strong cravings. In extreme cases, or at the request of participants, researchers immediately seek the assistance of clinical staff to intervene with appropriate clinical management. Training sessions are immediately discontinued in the event that a participant expresses distress or intense craving during a session. If this occurs, with the participant’s permission, assistance is sought from the clinical staff, and their continued participation is subject to review. Any reports of severe cravings or distress are recorded to monitor the safety of this intervention, and participants are reminded that they may withdraw their participation at any time without adverse consequences. At the end of phone follow-ups, participants are offered the contact details of a free 24-h telephone and web-based alcohol and drug counselling and referral service in case the follow-up questionnaires have triggered distress or cravings.

CBM has already shown promise as an adjunctive treatment to psychological counselling in people with an AUD. The present trial is the first multisite trial of CBM delivered as a stand-alone intervention (i.e. not delivered alongside psychotherapeutic interventions) during withdrawal treatment. Since withdrawal is a time of heightened neuroplasticity, when cognitive interventions may have increased effects, we hypothesise that this intervention will help prevent early relapse, generating cost savings for the healthcare system and extending opportunities for long-term recovery for people with AUDs.
